# Pre-eruptive intracoronal resorption in a 10-year-old girl: a review and case report

**DOI:** 10.3389/froh.2025.1624190

**Published:** 2025-07-24

**Authors:** Laresh Naresh Mistry, Shreyas Neelkanthan, Anusree Basu

**Affiliations:** Department of Pediatric and Preventive Dentistry, Bharati Vidyapeeth (Deemed to be University) Dental College and Hospital, Navi Mumbai, India

**Keywords:** PEIR, occult caries, deciduous (milk) teeth, pediatric dental care, intracoronal resorption

## Abstract

Pre-Eruptive Intracoronal Resorption (PEIR) is a pathological condition characterized by the resorption of dental tissues within the crown of an unerupted tooth. It is typically asymptomatic and is most often identified incidentally during radiographic examinations. The exact etiology of PEIR remains unclear, although various theories have been proposed, including developmental disturbances, trauma, and genetic factors. This article aims to provide a comprehensive review of PEIR, following the Case Report (CARE) checklist, to enhance understanding and awareness of this dental anomaly.

## Introduction and background

Pre-Eruptive Intracoronal Resorption (PEIR) is a developmental defect characterized by the presence of radiolucent lesions within the dentin, just beneath the Dentin-Enamel Junction (DEJ), occurring before the tooth erupts into the oral cavity. A related but distinct phenomenon is “hidden caries,” also known as “occult caries,” which primarily affects the occlusal surfaces of teeth (1). These lesions often go unnoticed during routine clinical examinations due to the enamel appearing intact on visual inspection. However, radiographic evaluation typically reveals radiolucent areas within the dentin, indicating underlying decay. It is important to differentiate PEIR and occult caries from various enamel defects such as hypoplasia, hypocalcification, hypomineralization, fluorosis, and Molar Incisor Hypomineralization (MIH), which differ significantly in both etiology and clinical appearance. For instance, enamel hypoplasia is a quantitative defect caused by incomplete enamel formation, resulting in visible pits, grooves, or areas of missing enamel, often associated with systemic illness or trauma during tooth development (2). Hypocalcification is a qualitative defect caused by insufficient calcium deposition, resulting in enamel that is soft, chalky, and more prone to decay. Hypomineralization refers to poor mineral content in the enamel, leading to teeth that are weak, porous, and discolored. Dental fluorosis results from excessive fluoride exposure during enamel formation, leading to diffuse opacities or mottling, with severity ranging from mild discoloration to significant structural defects. MIH affects the first permanent molars and incisors, making them more susceptible to rapid enamel breakdown and heightened sensitivity. Unlike fluorosis, MIH lesions are asymmetrical. Proper diagnosis and appropriate management of these conditions are essential to prevent functional and esthetic complications. Research indicates that the prevalence of intracoronal resorption ranges from 2% to 8% by subject and 0.6% to 2% by tooth (3). Permanent teeth (molars and premolars) are most affected by PEIR (4). Only one case of PEIR has been reported in the primary dentition (5). PEIR presents with no clinical diagnostic signs. The most effective method for detecting PEIR is pre-eruptive radiographic imaging, with Orthopantomograms (OPGs) being the most commonly used routine assessment tool. It is advised to thoroughly examine the crowns of all unerupted teeth radiographically to detect such lesions.

## Case report

A 10-year-old female patient presented to the Department of Paediatric and Preventive Dentistry at a private dental hospital with the chief complaint of a decayed tooth accompanied by pain in the lower right back region of the jaw for the last 4 months. When the patient presented to our clinic, an emergency access opening had already been performed on tooth 46 at a private dental clinic 3 weeks prior; however, no other prior oral or dental screening had been conducted, including through school or community programs. The patient reported a history of a cavity in the lower right back region of her jaw 6 months ago. Four months ago, she began experiencing pain associated with food lodgment, which aggravated during mastication and was relieved only by medication. A history of nocturnal pain was also noted. Medical, family, and other history were non-contributory, with no history of consanguineous marriage. Although genetic testing was advised, the patient's mother declined to proceed with it. The patient was fairly built, had a normal gait, and showed no apparent disabilities. Palpable submandibular lymph nodes were noted on the left side. At the first visit, the patient’s pain was measured using the Visual Analog Scale (VAS), with a recorded score was 6, indicating severe pain. The Caries Risk Assessment for Treatment (CRAFT) score was also assessed, and dietary recommendations were provided to the patient.

### Clinical findings

**Figure 1:**
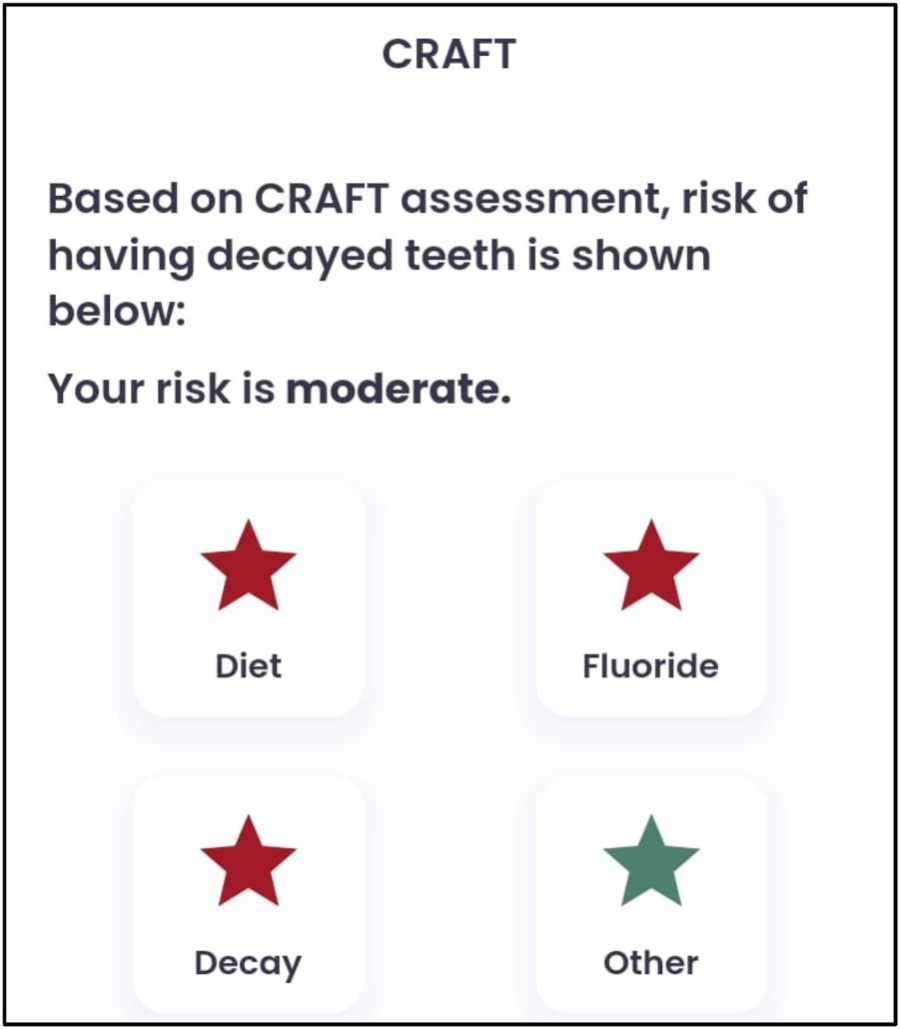
CRAFT score.

All the teeth in this report have been named as per Federation Dentaire Internationale (FDI) system. On clinical examination (Figure 1), the teeth present were 16, 55, **^^^**14, 53, 12, 11, 21, 22, 63, 64, 65, and 26 in the upper arch and 46, **^^^**45, **^^^**44, 83, 42, 41, 31, 32, 73, **^^^**34, 75, and 36 in the lower arch.

### Diagnostic investigation

**Figure 2:**
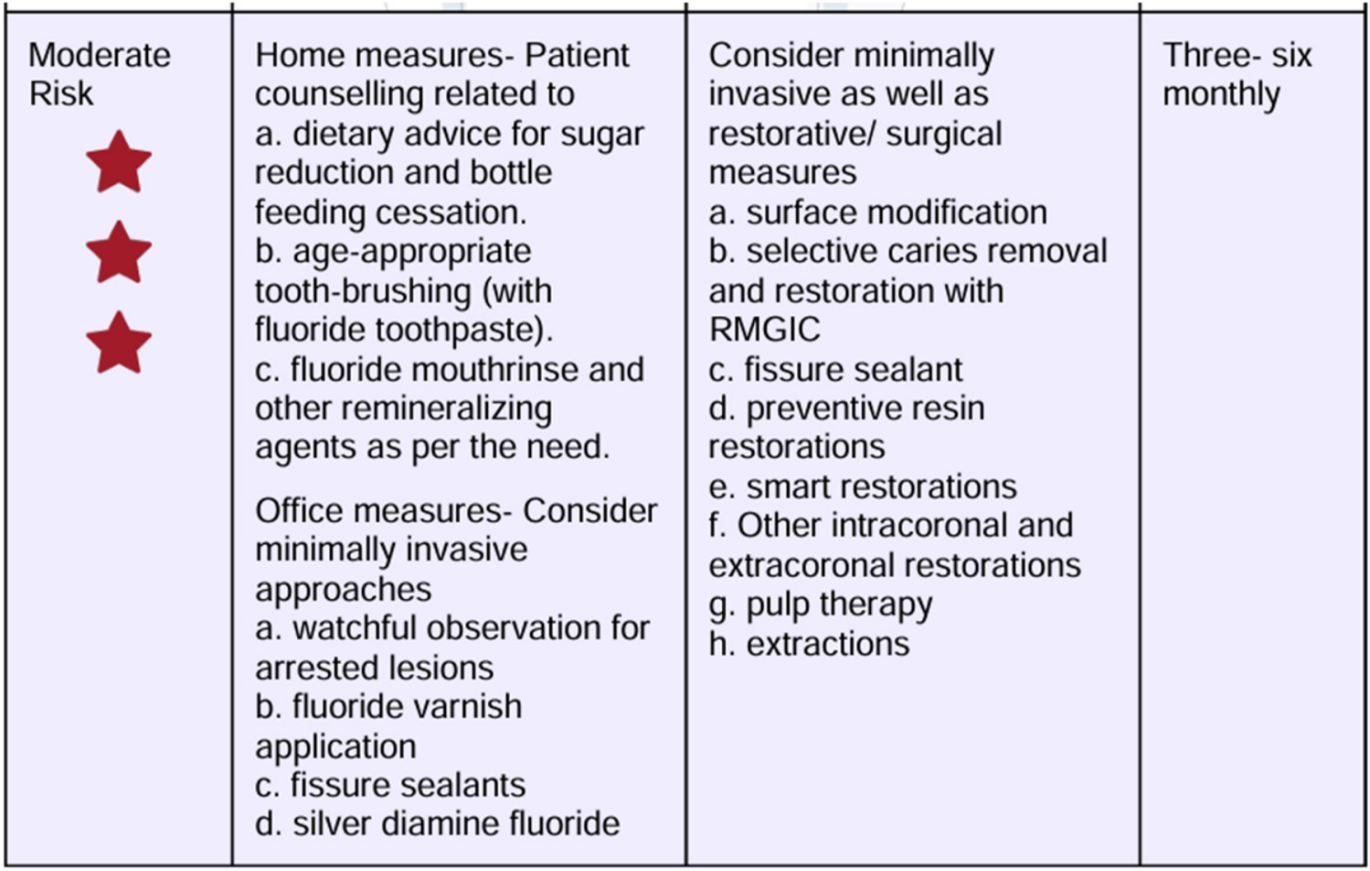
Dietary recommendations based on the CRAFT score.

An OPG was taken (Figure 2). On the basis of radiographic examination, we arrived at the following diagnoses (Table 1):

• Tooth 46: chronic irreversible pulpitis;
• Teeth 63, 16, and 26: enamel caries;
• Teeth 54, 55, 64, 65, 74, and 75: pre-shedding resorption;
• Tooth 36: dentinal caries; and
• Teeth 14, 15, 27, 33, 37, 44, 45, and 47: pre-eruptive intracoronal resorption.

**Figure 3:**
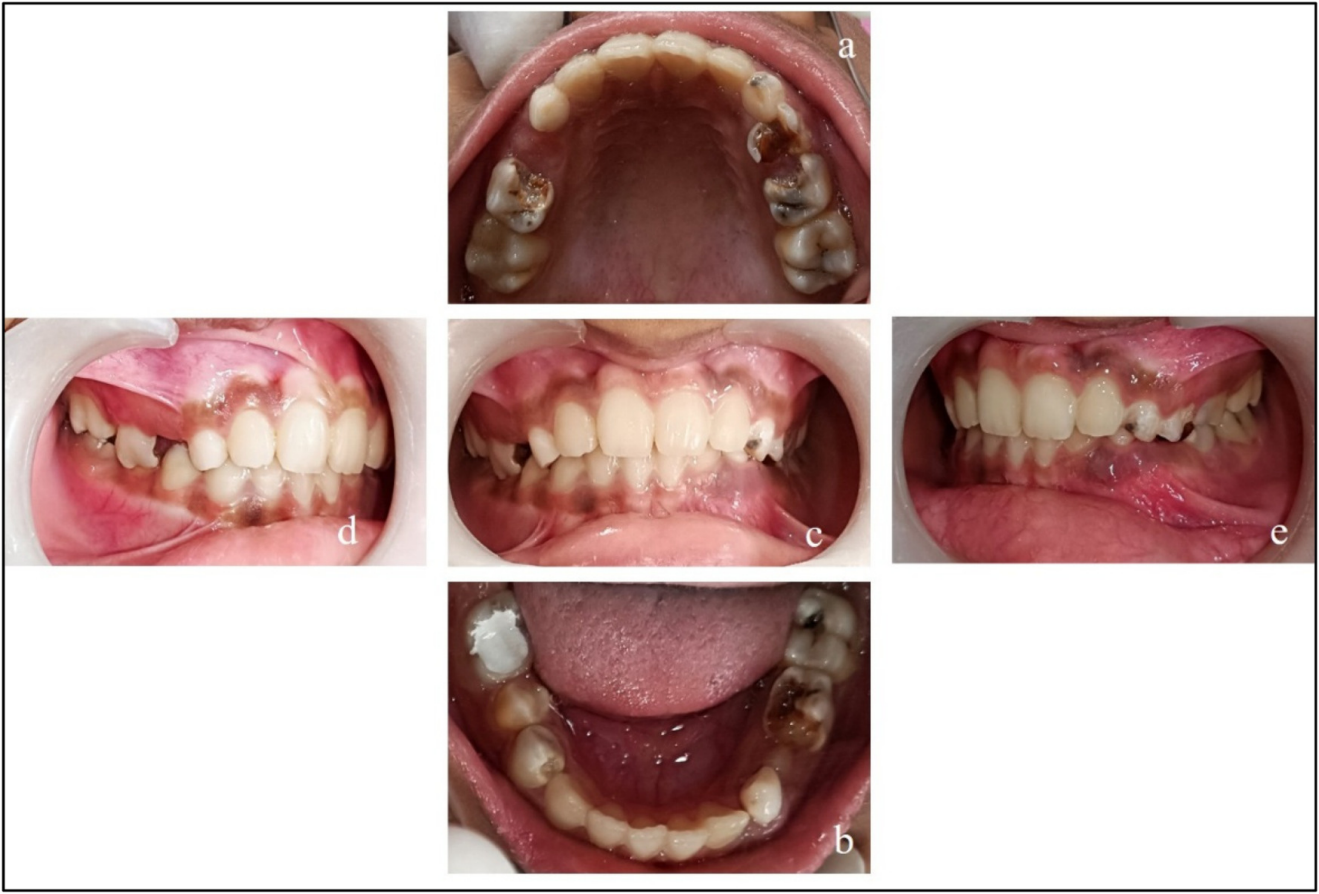
Pre-treatment records. **(a)** Maxillary arch; **(b)** mandibular arch; **(c)** at occlusion; **(d)** right lateral occlusion; and **(e)** left lateral occlusion.

**Figure 4:**
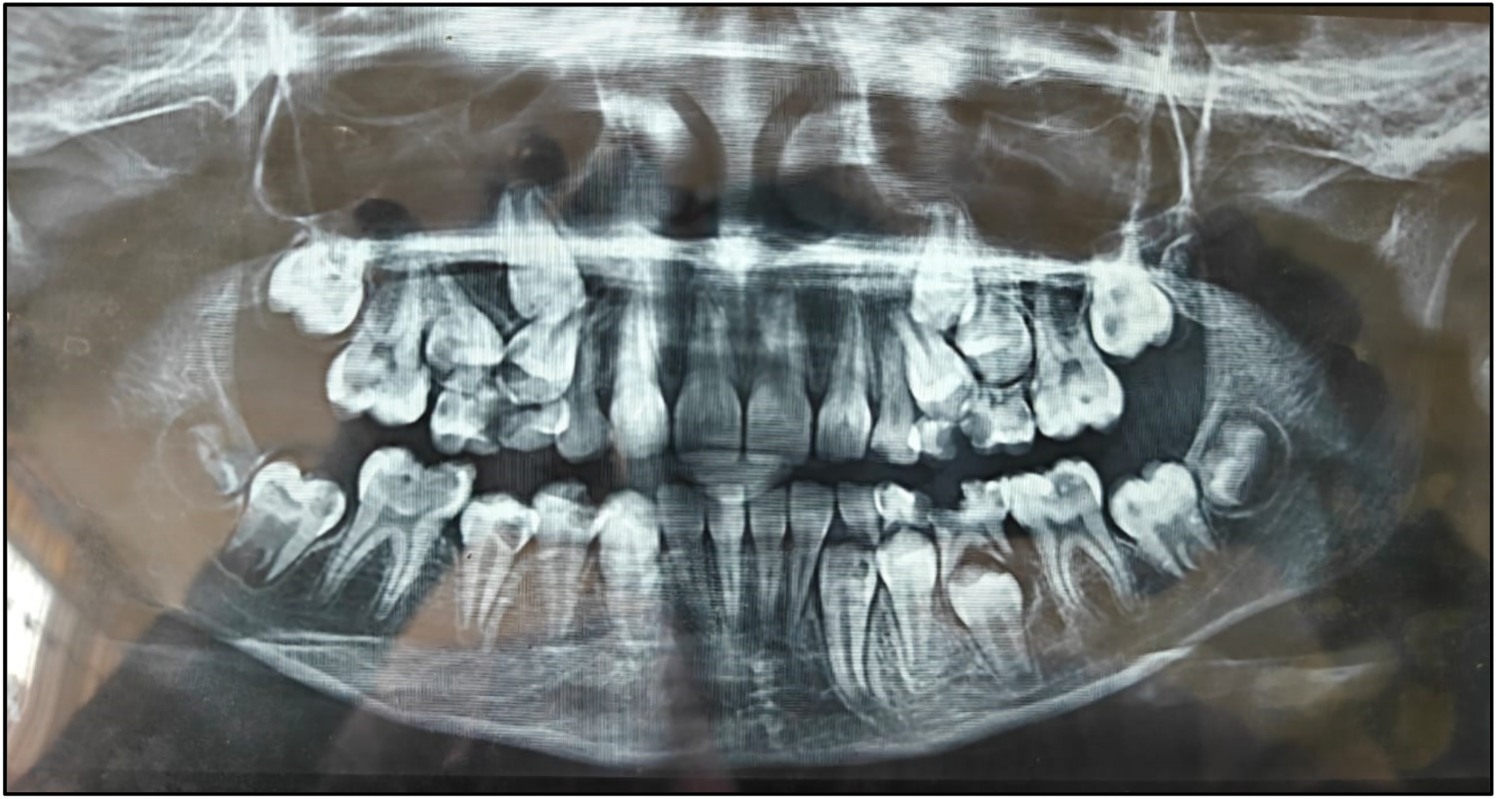
Pre-treatment OPG.

**Table 1:** Diagnosis.

Tooth no.	Clinical presentation
46	Occlusal caries involving enamel, dentine, and pulp
63	Mesio-proximal caries involving enamel
16	Pit and fissure caries involving enamel
26	Pit and fissure caries involving enamel
54, 55, 64, 65, 74, 75	Multi-surface caries with pre-shedding resorption
36	Deep occlusal caries involving enamel, dentin, and approaching pulp

Nolla staging was performed for the teeth affected by PEIR (Table 2).

**Table 2:** Nolla's staging of individual tooth.

Tooth number	Nolla stage
14	Stage 8
15	Stage 8
27	Stage 7
33	Stage 9
37	Stage 8
44	Stage 9
45	Stage 9
47	Stage 8

The differential diagnosis revealed enamel hypoplasia, fluorosis, hypomineralization, incipient enamel caries, and pre-eruptive intracoronal resorption.

The provisional and final diagnosis arrived at was Pre-Eruptive Intracoronal Resorption.

### Therapeutic intervention

The treatment for PEIR involved surgical removal of the caries, carried out in the following steps (Tables 3–5):

Step 1: Excavation of caries using a round bur [Mani BR 31 carbide round bur (Mani Inc., Tochigi, Japan)] under complete isolation.
Step 2: Placement of Biodentine™.
Step 3: Restoration using Light-Cure Glass Ionomer Cement (LC-GIC).

**Table 3:** Treatment plan.

Treatment plan		
Preparatory phase	• Oral prophylaxis • Caries control	Home measures • Increase intake of fruits and vegetables • Avoid sugary foods and carbohydrates • Brush twice daily with 1,000 ppm fluoridated toothpaste • Use of fluoride mouthrinse (Amflor)—5 ml once a day Office measures • Oral prophylaxis • Diet counseling based on CRAFT assessment
Restorative phase	• Restoration with teeth 16, 26, 63, 36, 44, 45, 14, 15, and 33	• Composite restoration—16, 26, 63 • Biomimetic liner + GIC (light-cure) restoration—36 • Biomimetic liner + GIC (light-cure) restoration—44, 45 • Biomimetic liner + composite (Giomer) restoration—14, 15, 33
Surgical phase	• Extraction with teeth 54, 55, 64, 65, 74, and 75	Extraction under local anesthesia (2% lignocaine hydrochloride) with adrenaline (1:80,000)
	• Root canal treatment with tooth 46 • Stainless Steel Crown (SSC) with teeth 36 and 46	Root canal treatment under local anesthesia (2% lignocaine hydrochloride) with adrenaline (1:80,000)
Orthodontic phase	Arch expansion and fixed mechanotherapy	The patient was explained the need for orthodontic treatment, but the patient was reluctant because of subjective fear and preferred to reconsider it upon completion of the other planned treatments

**Table 4:** Treatment objectives.

Steps	Objective
Step 1: Caries excavation using a round bur under complete isolation	To retain more tooth structure
Step 2: Placement of Biodentine™ (biomimetic material)	To induce a positive pulpal response
Step 3: Restoring using LC-GIC	All restorations are inferior; final full coverage treatment after eruption of 7 s, with the establishment of occlusion

**Table 5:** Visit-wise treatment plan.

Visit	Treatment plan
Visit 1	• Oral prophylaxis was performed • CRAFT assessment and diet recommendations were given • Access opening, working length determination, and biomechanical preparation were completed for tooth 46 using hand k-files (#10–35) under local anesthesia (2% lignocaine hydrochloride) with adrenaline (1:80,000)
Visit 2	• Obturation was completed for tooth 46. The patient was advised to return for follow-up after 3 weeks, to be followed by placement of a SSC on tooth 46 • Caries excavation and composite restoration were done on teeth 16 and 26 • Tooth 55 was extracted under local anesthesia (2% lignocaine hydrochloride) with adrenaline (1:80,000) using the local infiltration technique
Visit 3	• Caries excavation and composite restoration were done on tooth 63 • Teeth 64 and 65 were extracted under local anesthesia (2% lignocaine hydrochloride) with adrenaline (1:80,000) using the local infiltration technique
Visit 4	• Caries excavation and Biodentine™ + LC-GIC restoration were done on tooth 36. The patient was advised to return for follow-up after 4 weeks for evaluation of tooth 36 • Follow-up was done for tooth 46. Tooth preparation for an SSC was done, and tooth 46 cementation was done using glass ionomer cement (type I)
Visit 5	• Caries excavation and Biodentine™ + LC-GIC restoration were performed on teeth 44 and 45. The patient was advised to return for follow-up after 4 weeks for evaluation of teeth 44 and 45 (Figure 3)
Visit 6	• Follow-up was conducted for tooth 36. Tooth preparation for an SSC was completed, and tooth 36 cementation was done using glass ionomer cement (type I) • Tooth 75 was extracted under local anesthesia (2% lignocaine hydrochloride) with adrenaline (1:80,000) using inferior alveolar nerve block, lingual nerve block, and long buccal nerve block techniques
Visit 7	• Caries excavation and Biodentine™ + composite (Giomer) restoration were performed on tooth 15 • Gingival polyp cauterization was performed on tooth 14. Temporary restoration was done using Zinc Oxide Eugenol (ZnOE) cement. The patient was advised to return for follow-up after 1 week for evaluation of tooth 14 (Figure 4). Cauterization of the dental pulp in relation to tooth 14 was performed to control bleeding from the inflamed papillary gingiva. A temporary ZnOE cement was placed to maintain space for the subsequent placement of the definitive restoration
Visit 8	• Caries excavation and Biodentine™ + composite (Giomer) restoration were done on tooth 14 • The patient was recalled after 3 months for follow-up.
Visit 9	• The patient reported for follow-up after 3 months (Figures 5–7). Oral prophylaxis was done. An OPG was taken • Caries excavation and composite (Giomer) restoration were performed on tooth 33

### Follow-up and outcomes

**Figure 5:**
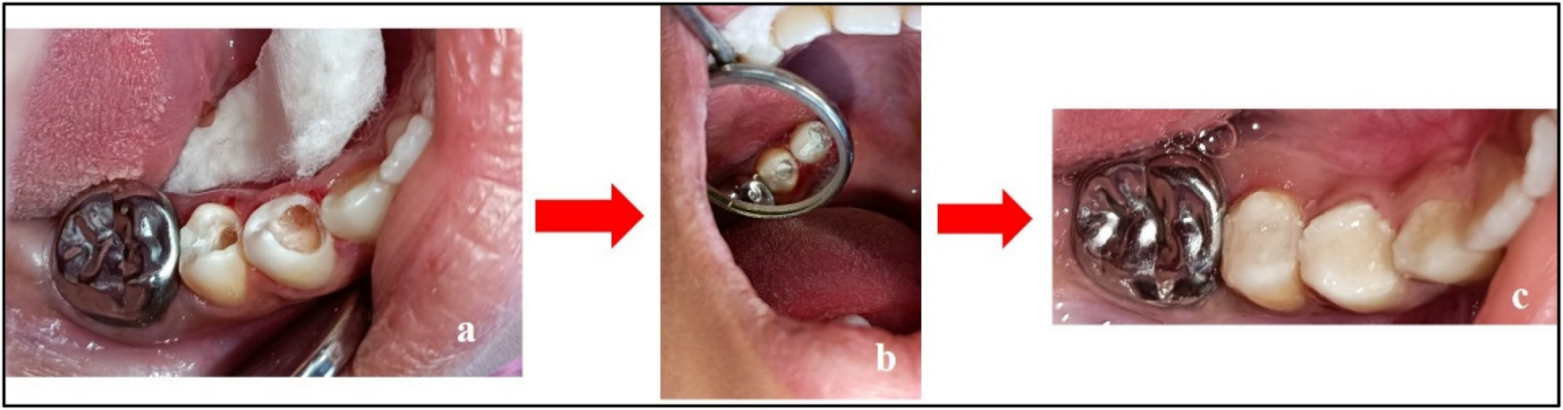
Treatment done w.r.t. teeth 44 and 45. **(a)** Caries excavation completed; **(b)** placement of Biodentine™; and **(c)** restoration using LC-GIC.

**Figure 6:**
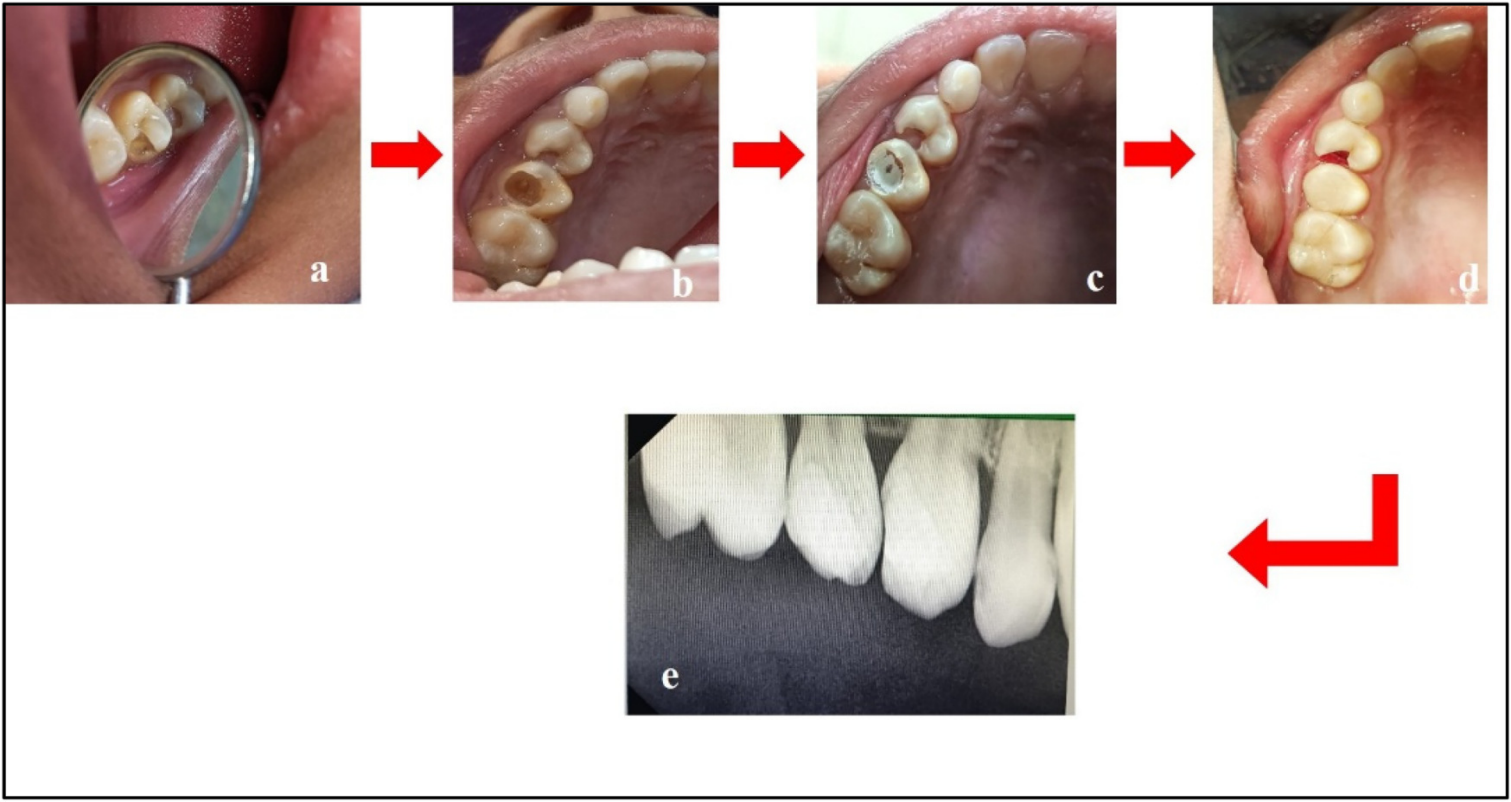
Treatment done w.r.t. teeth 14 and 15. **(a)** Pre-treatment; **(b)** caries excavation done w.r.t. tooth 15; **(c)** placement of Biodentine™ w.r.t. tooth 15; **(d)** composite restoration w.r.t. tooth 15 and cauterization of gingival polyp w.r.t. tooth 14; **(e)** post-treatment radiograph w.r.t. teeth 14 and 15.

A meticulous post-treatment follow-up was conducted. During the initial follow-up visit, an OPG was advised (Figure 5), which revealed a radiolucency in the region of teeth 13 and 14. To further evaluate and rule out any underlying pathology, a Cone Beam Computed Tomography (CBCT) scan of the 13, 14, 15 region was advised (Figure 8). CBCT findings revealed no pathological changes in the specified region; however, transposition of teeth 13 and 14 was noted (Figure 6).

**Figure 7:**
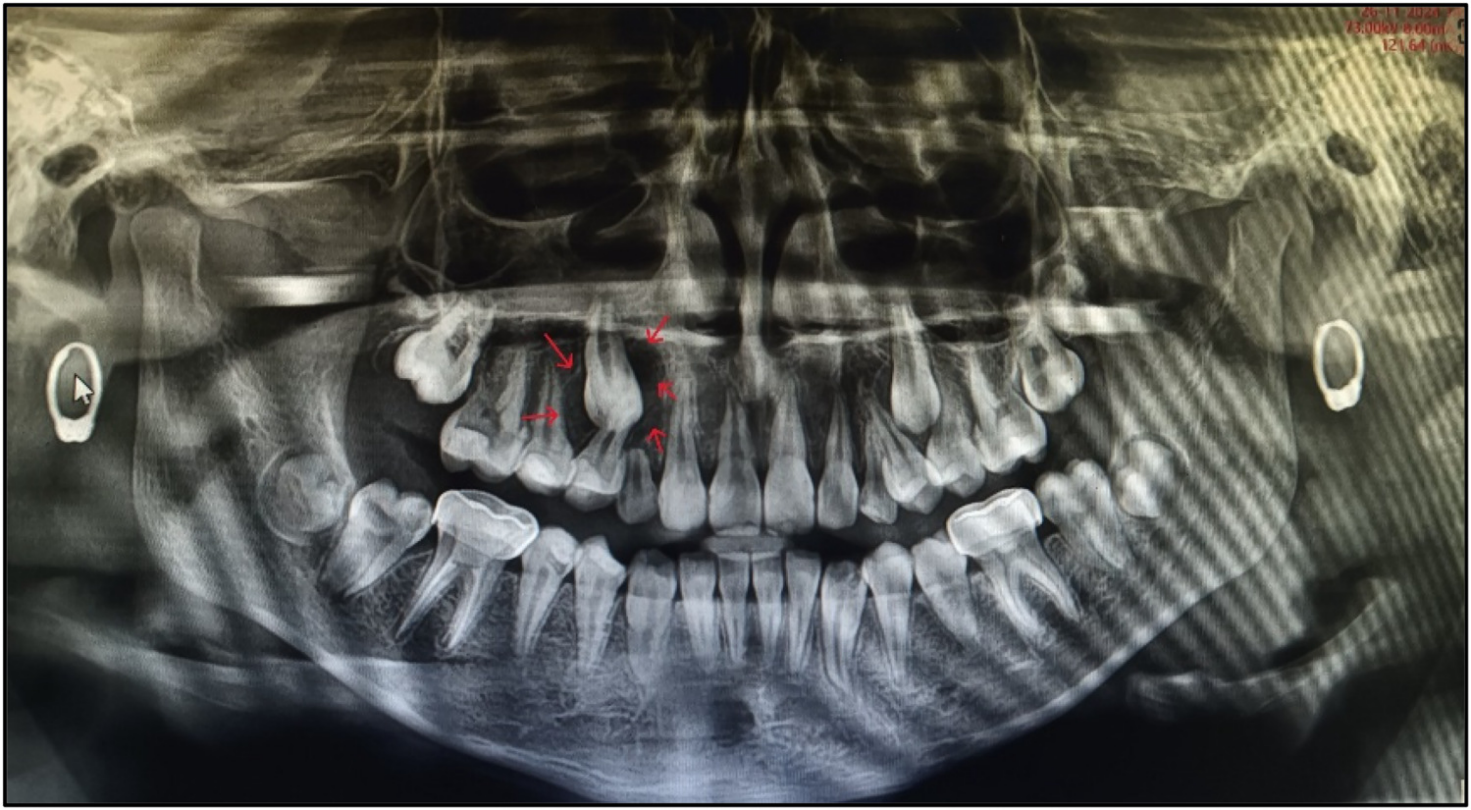
Post-treatment first follow-up OPG.

**Figure 8:**
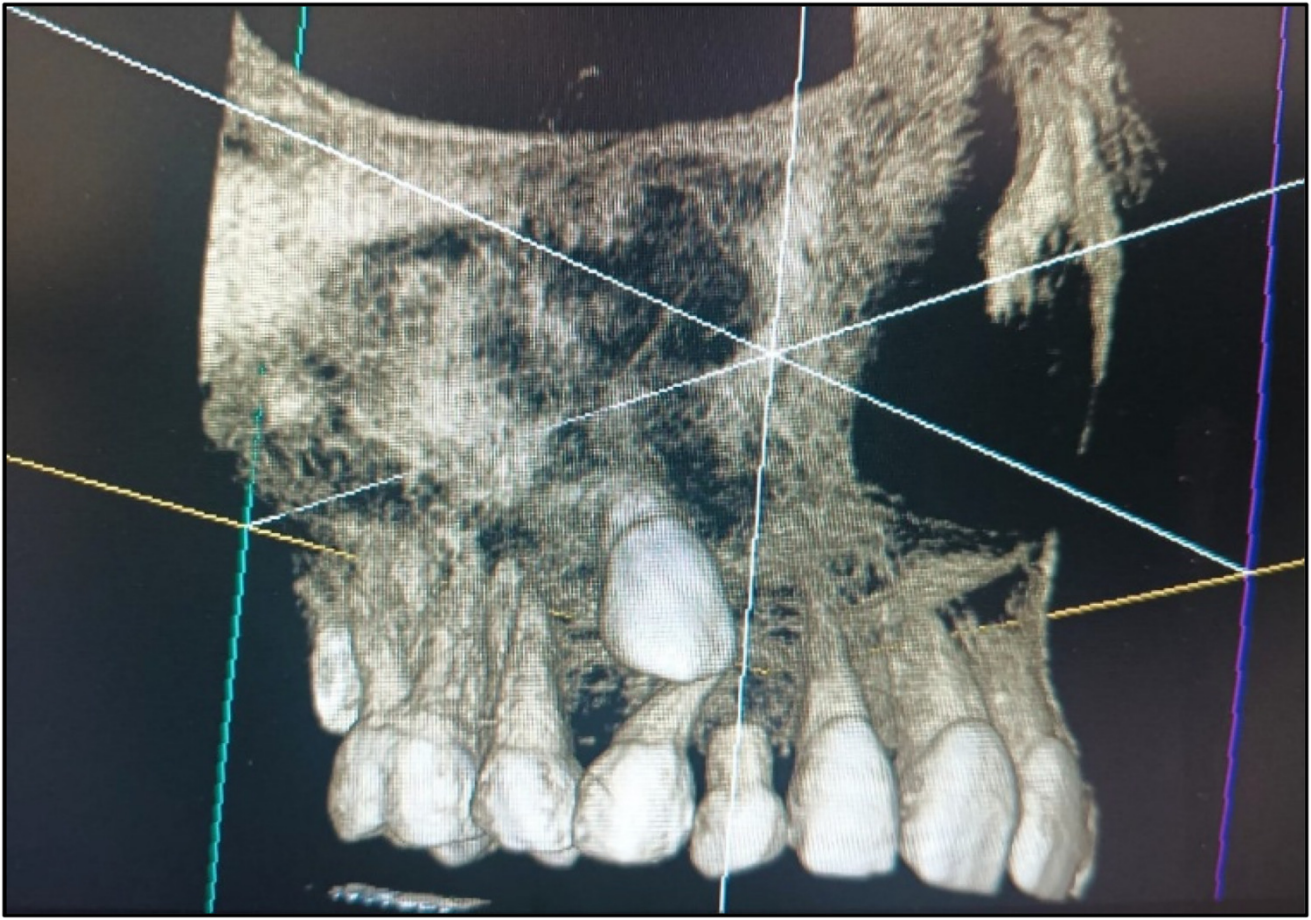
CBCT w.r.t. the 13, 14, 15 region.

**Figure 9:**
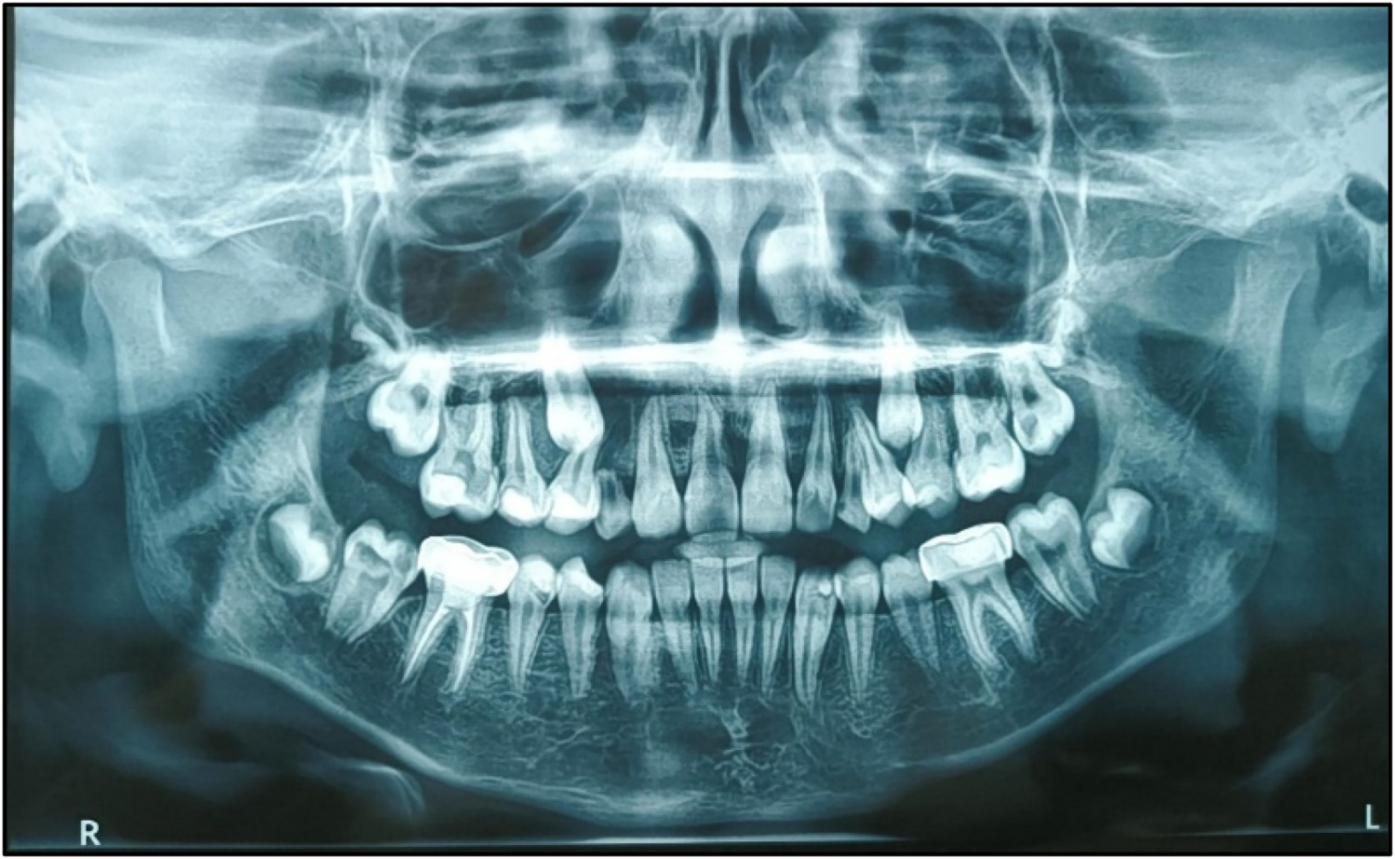
Post-treatment second follow-up OPG.

Follow-up is a critical component in the management of such cases. A 3-monthly follow-up protocol was implemented for the teeth affected by PEIR until complete eruption, followed by appropriate treatment. Subsequent follow-ups were scheduled every 3 months, tailored to the patient's individual caries risk assessment (as per CRAFT recommendations) (Figure 9).

The chief complaint was resolved, pain was relieved, and mastication improved. The patient and guardian expressed satisfaction with the treatment and appreciated the early intervention, which prevented further complications. However, the patient was unable to adhere to regular follow-up visits due to residence relocation.

## Discussion

To date, 10 reports in the literature have documented the incidence of PEIR. This report is the first to identify multiple teeth affected by PEIR. Existing literature indicate that PEIR exhibits no significant gender predilection and occurs equally across different geographical regions and racial groups. In terms of arch involvement, mandibular teeth demonstrate a higher incidence of PEIR compared to maxillary teeth. Within the mandibular arch, the first premolars are most frequently affected, whereas in the maxillary arch, canines show the highest prevalence of PEIR lesions (6).

Routine radiographic examinations, particularly panoramic radiographs, play a crucial role in detecting PEIR, as these lesions are often identified incidentally. A study analyzing 3,143 orthodontic patients reported a PEIR prevalence of 1.56%, with a higher occurrence in the mandibular arch. Early detection is essential for timely intervention, mitigating the risk of lesion progression and associated complications (7).

Early diagnosis and timely intervention of teeth affected by PEIR have always posed a great challenge for dentists (8). In this case, the diagnosis was made before tooth eruption, confirming it as PEIR, because unerupted teeth are not invaded by bacteria and thus a carious process cannot occur.

Some studies suggest a potential association between PEIR in permanent teeth and conditions in primary teeth. For instance, one study observed that advanced PEIR defects (grades 2 and 3) were more common in patients with missing primary teeth or those with primary teeth exhibiting periapical lesions (9). While some evidence suggests a potential association between PEIR in permanent teeth and conditions in primary teeth, the relationship is not yet definitive. Further comprehensive studies are needed to establish a clear association between PEIR in permanent teeth and anomalies in primary teeth.

Managing PEIR presents several challenges for dentists, primarily due to its asymptomatic nature and pre-eruptive occurrence, making early detection difficult. Since PEIR lesions are detectable only through radiographic imaging, particularly OPG or CBCT scans, routine imaging is essential for accurate diagnosis (10).

Another challenge is differentiating PEIR from caries, as both appear radiolucent. However, unlike caries, PEIR lesions are non-cavitated before eruption. If left untreated, PEIR can lead to structural weakening of the tooth, making the tooth prone to fracture or infection after eruption (10).

The OPG is commonly used for initial screening. CBCT offers 3D imaging to assess the extent of resorption. Periapical radiographs are useful when the teeth begin to erupt. Although rarely used, transillumination and ultrasonography may assist in assessing enamel defects or tooth density (11). Although not routinely used, histopathological investigations can help confirm the diagnosis in extracted teeth by identifying odontoclastic activity (12).

In this case, the diagnosis of PEIR was made incidentally on an OPG. Clinical management of PEIR is very complex in a developing dentition. To arrive at a treatment plan, dental practitioners must consider several factors. Clinical management of a tooth affected by PEIR defects depends on the extent of decay/defect and the rate of its progression at the time of diagnosis. Various treatment options suggested in the literature include restoration before eruption, restoration soon after eruption, and extraction of the affected tooth. If the lesion is extensive, early restorative intervention may be required before complete tooth eruption, which can be challenging due to limited access (13). In cases where pulp is involved, vital pulp therapy or root canal treatment may be needed (3).

In this case, we chose to treat conservatively because there was no communication between the lesion and the pulp. Mineral Trioxide Aggregate (MTA) or Biodentine™ is the material of choice for treating such cases. For this case, the chosen material was Biodentine™, which was placed over the decay and followed by LC-GIC as the coronal filling material. In cases of extensive resorption, extraction may be the only viable option, requiring careful treatment planning to address prosthetic or orthodontic needs.

A conservative approach with regular clinical and radiographic follow-up is recommended for non-progressive PEIR lesions. The literature suggests follow-up intervals of every 6 to 12 months. Continuous monitoring is necessary to evaluate any changes in size or severity of PEIR lesions. Research findings indicate that approximately 89.1% of PEIR lesions remain stable over a mean follow-up period of 36.4 months. However, given the potential for lesion progression, periodic radiographic assessment is recommended to inform appropriate management strategies (7). Therefore, comprehensive risk assessment and long-term follow-up are fundamental in the management of PEIR. Early detection through radiographic screening and regular monitoring enables timely intervention, thereby preventing complications and ensuring optimal treatment outcomes.

## Conclusion

The main “takeaway” lesson is that clinical diagnosis of PEIR is very difficult. A panoramic radiograph (orthopantomogram), along with thorough evaluation and detailed clinical examination, is essential for diagnosing this condition, which may have detrimental long-term consequences. If left undiagnosed at an early stage, teeth affected by PEIR may become non-restorable within a few months after eruption.

## Data Availability

The original contributions presented in the study are included in the article/Supplementary Material, further inquiries can be directed to the corresponding author.
